# Supplementary material for: The Pandora’s Box of Frailty Assessments: Which Is the Best for Clinical Purposes in TAVI Patients? A Critical Review
Source: J Clin Med. 2021 Sep 29;10(19):4506. doi: 10.3390/jcm10194506 (PMC8509314; doi:10.3390/jcm10194506)
Supplement: Supplementary file 1 [file jcm-10-04506-s001.zip › jcm-1368780-supplementary.pdf]

Table S1. Summary of the carachterists of included studies and assessments of Bias (Quality in Prognosis Studies [A]; Newcastle-Ottawa Scale [B])

Author	a. Country<br>b. Study name<br>c. Registry/database	Study design<br>*(Observational prospective : Op/ retrospective: Or)*	Study population<br>*a: Sample size*<br>*c: Male (%)*	 *b: Age*<br>*d: No. frail patients (%)*	Frailty assessment	Follow-up<br>*(Short: ≤ 30-day; Intermediate: > 30-day to < 1-year; Long: ≥ 1-year)*	Overall risk of Bias<br>QUIPS [A]	 NOS [B]
Abdul-Jawad Altisent et al. 2017	a. Canada	single center/Op	a: 305<br>c: 44	b: 79 ± 9<br>d: n.a.	○ Gait speed (6MWT)	Long-	Low	9
Abramowitz et al. 2016	a. USA	single center/Op	a: 805<br>c: 60.1	b: 82 ± 8.8<br>d: 279 (34.7)	○ Composite index (gait speed, serum albumin, handgrip strength, IADL)<br>○ BMI	Long-	Moderate	7
Afilalo et al. 2010	a. USA, Canada	multicenter/Op	a: 131<br>c: 46	b: 75.8 ± 4.4<br>d: 60 (46)	○ Gait speed (5-mWT)	Short-	Low	7
Afilalo et al. 2017	a. USA, Canada, France<br>b. FRAILTY-AVR	multicenter/Op	a: 646<br>c: 59	b: 82 (median)<br>d: Fried (49)<br>Fried+ (64)<br>CFS (35)<br>SPPB (74)<br>Bern-scale (60)<br>Columbia (59)<br>EFT (37)	○ Fried (5-domains)<br>○ Fried+ (+MMSE)<br>○ CFS<br>○ SPPB<br>○ Bern-scale<br>○ Columbia (4-domains)<br>○ EFT	Short-<br>Long-	Moderate	9
Alfredsson et al. 2016	a. USA<br>c. STS/ACC TVT	multicenter/Op	a: 8.039<br>c: 48.6	b: 84 (79-88)<br>d: 6.100 (75.88)	○ Gait speed (5-mWT)	Short-	Moderate	6
Arnold et al. 2018	a. USA<br>c. STS/ACC TVT	multicenter/Op	a: 21.661<br>c: 51.6	b: ≥75 (79.3%)<br>d: n.a.	○ Gait speed (5-mWT)	Short-	Moderate	5
Assmann et al. 2016	a. Netherlands	single center/Op	a: 89<br>c: 43	b: 80.4 ± 6.3<br>d: n.a.	○ Gait speed/TUG<br>○ MMSE<br>○ BADL<br>○ IADL (Lawton index)<br>○ MNA<br>○ Composite index (from all above)	Short-	High	4
Berkovitch et al. 2020	a. Israel	single center/Or	a: 2.608<br>c: 45	b: 82 ± 7<br>d: n.a.	○ Serum albumin	Long-	Moderate	7
Bogdan et al. 2016	a. Israel	single center/Or	a: 150<br>c: 40	b: 81 ± 6<br>d: 79 (53)	○ Serum albumin	Long-	Moderate	9
Boureau et al. 2017	a. France	multicenter/Op	a: 150<br>c: 56	b: 83.7 ± 4.6<br>d: BMI: 18 (12)<br>TUG: 83 (55.3)	○ CIRS-G<br>○ TUG<br>○ MMSE<br>○ FAB<br>○ BMI<br>○ IADL (Lawton index)<br>○ mini-GDS<br>○ CGA-scale (from all above)	Intermediate-	Low	6
Bureau et al. 2017	a. France	single center/Op	a: 116<br>c: 49.1	b: 86.2 ± 4.2<br>d: n.a.	○ SPMSQ<br>○ ESS<br>○ ADL (Katz index)<br>○ IADL (Lawton index)	Intermediate-<br>Long-	Moderate	7

					o CIRS index<br>o MNA-SF<br>o MPI scre (from all above)			
Capodanno et al. 2014	a. Italy<br>b. OBSERVANT	multicenter/Op	a: 1.256<br>c: 42.4	b: 81.9 ± 5.9<br>d: Level 1: 377 (20.1)<br>Level 2: 404 (21.5)<br>Level 3: 55 (2.9)<br>Level 2 or 3: 459 (24.4)	o GSS scale	Short-	Moderate	8
Chauhan et al. 2016	a. USA	single center/Or	a: 342<br>c: 47.7	b: 81.8 ± 7.5<br>d: Gait speed: 231 (67.54)<br>Handgrip: 294 (85.96)<br>Albumin: 80 (23.39)<br>Katz index: 81 (23.68)<br>Index ≥ 3/4: 104 (30.41)	o Gait speed (5-mWT)<br>o Serum albumin<br>o Katz index<br>o Handgrip strength (dynamometer)<br>o Composite index (from all above)	Long-	Moderate	6
Cockburn et al. 2015	a. UK<br>c. UK CCAD	single center /Or	a: 312<br>c: 53.2	b: 81.2 ± 7<br>d: n.a.	o EuroSCORE (mobility)<br>o Brighton Mobility Index<br>o NYHA-scale<br>o Karmofsky Performance Scale<br>o Katz index<br>o CSHA	Sort-<br>Long-	Moderate	5
Codner et al. 2015	a. Israel	single center/Op	a: 360<br>c: 43.6	b: 82.1 ± 6.9<br>d: n.a.	o Composite index (gait speed, ADL, serum albumin, need for oxygen, cognitive status, patient's apparence, clinician judgement)	Long-	High	5
Drudi et al. 2018	a. USA, Canada, France<br>b. FRAILTY-AVR	multicenter/Op	a: 723<br>c: 55	b: 83.5 ± 5.6<br>d: EFT: 254 (35.1)<br>SPPB: 282 (39)	o EFT<br>o SPPB	Short-<br>Long-	Low	7
Dvir et al. 2013	a. USA<br>b. PARTNER Trial	multicinter/Or	a: 1.108<br>c: 54.4	b: 82.7 ± 7.2<br>d: n.a.	o Gait speed (6MWT)	Long-	Low	8
Dziewierz et al. 2017	a. Poland	single center/Op	a: 148<br>c: 37.8	b: 82 (77-85)<br>d: Gait speed: 21 (14.2)<br>EMS: 8 (5.4)<br>CSHA: 17 (11.5)<br>Katz index: 19 (12.8)<br>Handgrip: 7 (4.7)<br>ISAR: 53 (35.8)	o Katz index<br>o EMS<br>o CSHA<br>o Gait speed (5-mWT)<br>o Handgrip strenght (hand grasp)<br>o ISAR scale	Long-	High	6
Eichler et al. 2017	a. Germany	multicenter/Op	a: 344<br>c: 44.5	b: 80.9 ± 5.0<br>d: Bern scale: 152 (45.8)	o MMSE<br>o TUG<br>o MNA<br>o BADL (Katz index)<br>o IADL (Lawton index)<br>o Pre-clinical mobility disability<br>o Bern scale (from all above)	Long-	Low	8
Ewe et al. 2010	a. Netherlands, Italy	multicenter/Op	a: 147<br>c: 42.8	b: 80 ± 7<br>d: 48 (32.6)	o Composite index (according to Fried 5-domains)	Long-	Moderate	8
Foldyna et al. 2018	a. USA	single center/Or	a: 403<br>c: 47.9	b: 82.5 ± 8.0<br>d: n.a.	o Psoas muscle area<br>o Visceral adipose tissue<br>o Subcutaneous adipose tissue	Long-	Moderate	6

Forcillo et al. 2017	a. USA	single center/Or	a: 361<br>c: 53.7	b: 82 (76-86)<br>d: n.a.	○ Serum albumin<br>○ Gait speed (5-mWT)<br>○ Handgrip strength (dynamometer)<br>○ Katz index	Short-<br>Long-	Moderate	4
Garg et al. 2017	a. USA	single center/Op	a: 152<br>c: 85	b: 83.3 ± 6.5<br>d: 76 (50)	○ Psoas muscle area	Long-	Low	8
González Ferreiro et al. 2019	a. Spain	multicenter/Or	a: 941<br>c: 34	b: 80.7 ± 6.5<br>d: 453 (48.1)	○ NRI	Long-	Moderate	6
Goudzwaard et al. 2018	a. Netherlands	single center/Op	a: 213<br>c: 47	b: 82 (78.2-85.6)<br>d: Lawton index: (43.2)<br>Katz index: (31.5)<br>TUG: (16.9)<br>MUST: (11.7)<br>MMSE: (34.7)<br>Erasmus score: 61 (28.6)	○ MMSE<br>○ Handgrip strength (dynamometer)<br>○ MUST<br>○ ADL (Katz index)<br>○ IADL (Lawton index)<br>○ Erasmus Frailty score (from all above)	Short-	Moderate	5
Green et al. 2015	a. USA<br>b. PARTNER Trial	multicenter/Or	a: 244<br>c: Frail 47/ Non-frail 55	b: Frail: 87.1 (82.7-90.3)<br>Non-Frail: 85.4 (79.4-89.5)<br>d: Katz index: 38 (35)<br>Index: 110 (45)	○ Gait speed (5-mWT)<br>○ Handgrip strength (dynamometer)<br>○ Serum albumin<br>○ ADL (Katz index)<br>○ Composite index (from all above)	Long-	Low	7
Green et al. 2013	a. USA<br>b. PARTNER Trial	multicenter/Or	a: 484<br>c: Unable: 45<br>Slow: 55<br>Fast: 67	b: Unable: 84.6 (79.1-88.9)<br>Slow: 85.9 (81.7-88.5)<br>Fast: 83.6 (78.3-87.6)<br>d: n.a.	○ Gait speed (6MWT)	Long-	Moderate	6
Green et al. 2012	a. USA	single center/Op	a: 159<br>c: 50	b: 86.2 ± 7.7<br>d: Index: 76 (47.7)	○ Gait speed (5-mWT)<br>○ Handgrip strength (dynamometer)<br>○ Serum albumin<br>○ ADL (Katz index)<br>○ Composite index (from all above)	Long-	Low	8
Grossman et al. 2017	a. Israel	single center/Or	a: 426<br>c: 43.2	b: 83.8<br>d: 192 (45)	○ Serum albumin	Long-	Low	7
Hermiller et al. 2016	a. USA<br>b. Medtronic CoreValve U.S. Pivotal Trial	multicenter/Or	a: 3.687<br>c: 53.7	b: 83.3 ± 7.8<br>d: BMI: 311 (8.4)<br>Weight loss: 411 (11.1)<br>Gait speed: 2.555 (82.4)<br>Handgrip: 2.489 (68.3)<br>Mobility: 722 (19.6)<br>Anemia: 674 (19.2)	○ Gait speed (5-mWT)<br>○ Handgrip strength (dynamometer)<br>○ BMI<br>○ Anemia requiring transfusion<br>○ Unintentional weight loss<br>○ Mobility (recent falls or wheelchair)	Short-<br>Long-	Low	7
Honda et al. 2019	a. Japan	single center/Or	a: 150<br>c:	b: High CONUT: 87 ± 5<br>Low CONUT: 86 ± 5<br>d: CONUT: 30 (20)	○ CFS<br>○ CONUT	Long-	Low	6
Huded et al. 2016	a. USA	single center/Or	a: 191<br>c: 51	b: 82.4 ± 9.2<br>d: 64 (33)	○ Composite index (gait speed, handgrip strength, ADL, unintentional weight loss)	Short-	Moderate	5

Kamga et al. 2013	a. Belgium	single center/Op	a: 30<br>c: 53	b: 86 ± 3<br>d: ISAR: 9 (30)<br>SHERPA: 11 (36.6)	o ISAR<br>o SHERPA	Short-<br>Long-	Moderate	5
Khan et al. 2019	a. Canada	single center/Op	a: 234<br>c: 59.4	b: 82.3 ± 6.7<br>d: 23 (9.8)	o SMARTIE	Short-	Moderate	6
Kiani et al. 2020	a. USA<br>c. STS/ACC TVT	multicenter/Or	a: 36.242<br>c: 52	b: 82.5 ± 6.9<br>d: n.a.	o Serum albumin<br>o Gait speed (5-mWT)<br>o Hemoglobin level	Short-<br>Long-	Low	7
Kleczynski et al. 2017	a. Poland	single center/Op	a: 101<br>c: 39.6	b: 81 (76-84)<br>d: Gait speed: 18 (17.8)<br>EMS: 8 (7.9)<br>CSHA: 17 (16.9)<br>Katz index: 18 (17.8)<br>Handgrip: 7 (6.9)<br>ISAR: 53 (52.5)	o Gai speed (5-mWT)<br>o Handgrip strength (hand grasp)<br>o Katz index<br>o EMS<br>o CSHA<br>o ISAR	Long-	Moderate	8
Kleczynski et al. 2018	a. Poland	single center/Op	a: 153<br>c: 35.9	b: 82 (77.5-85)<br>d: Gait speed: 21 (13.7)<br>EMS: 8 (16)<br>CSHA: 17 (34)<br>Katz index: 19 (12.4)<br>Handgrip: 7 (14)<br>ISAR: 44 (28.7)	o Gait speed (5-mWT)<br>o Handgrip strength (hand grasp)<br>o Katz index<br>o EMS<br>o CSHA<br>o Psoas muscle area<br>o Psoas muscle volume	Long-	Moderate	8
Kobe et al. 2016	a. Switzerland, Germany	multicenter/Op	a: 130<br>c: 50	b: 83.3 ± 4.8<br>d: FORECAST: 40 (30.7)	o FORECAST<br>o CFS<br>o Serum albumin	Short-	Low	8
Kofler et al. 2018	a. Austria, Germany	multicenter/Op	a: 1.076<br>c: 44	b: 83 (79-86)<br>d: n.a.	o Psoas muscle area	Short-<br>Long-	Low	8
Koifmann et al. 2015	a. USA	single center/Or	a: 567<br>c: Low-albumin: 79<br>Normal: 87	b: Low ablumin: 84 ± 8<br>Normal: 83 ± 7<br>d: 238 (50)	o Serum albumin	Long-	Moderate	6
Koifmann et al. 2016	a. USA	single center/Or	a: 491<br>c: 50	b: 83 ± 8<br>d: 43 (8.7)	o BMI	Long-	Moderate	8
Krishnan et al. 2019	a. USA	single center/Or	a: 381<br>c: 42.3	b: 82 ± 8<br>d: psoas level 151 (39.6)<br>paravertebral level 191 (50.1)	o Serum albumin<br>o mFI<br>o Psoas muscle area<br>o Paravertebral muscles area	Long-	Moderate	6
Kundi et al. 2019	a. USA<br>c. CMS MedPAR	multicenter/Or	a: 28.531<br>c: 53.6	b: 81.5 ± 8.1<br>d: 13.593 (47.6)	o Hospital Frailty Risk Score	Long-	Moderate	8
Kundi et al. 2018	a. USA<br>c. CMS MedPAR	multicenter/Or	a: 52.338<br>c: 51.9	b: 82.4 ± 8<br>d: 38.863 (66.6)	o John Hopkins Claims-based Frailty indicator	Long-	Moderate	8
Lee et al. 2019	a. South Korea	multicenter/Or	a: 412<br>c: 48.1	b: 78.7 ± 5.2<br>d: GNRI 227 (55.1)<br>CONUT 306 (74.3)	o GNRI<br>o CONUT	Long-	Moderate	6
Mamane et al. 2016	a. Canada, Germany<br>b. Montreal-Munich study	multicenter/Op	a: 208<br>c: 45	b: 80.7 ± 6.8<br>d: n.a.	o Psoas muscle area	Long-	Moderate	8

Martin et al. 2017	a. UK<br>c. UK TAVI	multicenter/Or	a: 6.339<br>c: 53.8	b: 81.3 (29-101)<br>d: n.a.	○ Katz index<br>○ CSHA<br>○ EuroSCORE (mobility)	Short-	Moderate	7
Martin et al. 2018	a. UK<br>c. UK TAVI	multicenter/Or	a: 2.624<br>c: 54.6	b: 81.2 ± 7.6<br>d: CSHA 1.043 (39.7)<br>Katz 846 (32.2)<br>EuroSCORE 591 (22.5)	○ Katz index<br>○ CSHA<br>○ EuroSCORE (mobility)	Short-<br>Intermediate-<br>Long-	Moderate	6
Michel et al. 2019	a. Germany	single center/Or	a: 1.731<br>c: 47.5	b: 81 (77-85)<br>d: n.a.	○ Psoas muscle area (cross-section)<br>○ Serum Albumin<br>○ Hemoglobin	Long-	Low	9
Miura et al. 2017	a. Japan<br>c. KMH	single center/Or	a: 112<br>c: 33.9	b: 84.5 ± 6.6<br>d: < 90 years 47 (54)<br>> 90 years 20 (83.3)	○ CFS	Long-	Moderate	6
Okoh et al. 2017	a. USA	single center/Op	a: 75<br>c: 35	b: 92 ± 2<br>d: 30 (40)	○ Frailty score (handgrip strength, gait speed, serum albumin, ADL)	Short-<br>Long-	Moderate	7
Okoh et al. 2019	a. USA<br>c. NBIMC	multicenter/Or	a: 117<br>c: 53	b: Group A: 69 ± 6<br>B: 81 ± 3<br>C: 89 ± 3<br>d: 29 (25)	○ Frailty score (handgrip strength, gait speed, serum albumin, ADL)	Long-	Low	7
Okuno et al. 2019	a. Japan	single center/Or	a: 95<br>c: 29.5	b: 84 (81-88)<br>d: n.a.	○ CONUT<br>○ PNI<br>○ GNRI	Long-	Moderate	6
Patel et al. 2019	a. USA	single center/Or	a: 431<br>c: 51	b: 81.0 ± 8.4<br>d: 74 (18)	○ Gait speed (5-mWT)<br>○ Serum albumin	Sort-	Low	7
Pighi et al. 2019	a. USA, Canada, France<br>b. FRAILTY-AVR	multicenter/Op	a: 759<br>c: 55.2	b: Women: 84.2 ± 5.3<br>Men: 82.8 ± 5.8<br>d: EFT 258 (40)<br>CFS n.a.<br>Columbia 453 (59.7)<br>Fried 335 (44)<br>SPPB 287 (37.7)	○ EFT<br>○ CFS<br>○ Columbia (4-domains)<br>○ Fried (5-domains)<br>○ SPPB	Long-	Moderate	8
Puls et al. 2014	a. Germany	single center/Op	a: 300<br>c: 34	b: 82.1 ± 5.3<br>d: 144 (48)	○ Katz index	Short-<br>Long-	Low	7
Rogers et al. 2018	a. USA	single center/Op	a: 544<br>c: 49	b: 81 ± 8.4<br>d: 242 (44)	○ Composite index (gait speed, BMI, serum albumin, ADL, handgrip strength)	Short-<br>Long-	Moderate	6
Saji et al. 2016	a. USA	single center/Or	a: 236<br>c: 57	b: 80.1 ± 8.7<br>d: n.a.	○ Psoas muscle area<br>○ Gait speed (5-mWT)	Short-<br>Intermediate-	High	6
Saji et al. 2019	a. Japan	single center/ Or	a: 455<br>c: 68	b: Group A: 85.8 ± 4.5<br>B: 83.6 ± 5.1<br>d: n.a	○ CFS	Long-	Moderate	5
Sathananthan et al. 2019	a. USA, Canada, France<br>b. FRAILTY-AVR	multicenter/Op	a: 755<br>c: 55	b: 83.5 ± 5.6<br>d: EFT ≥3: 254 (34)<br>EFT ≥5: 240 (32)<br>CFS n.a.	○ EFT<br>○ CFS<br>○ Gait speed (5-mWT)	Long-	Moderate	7

Schoenenberger et al. 2018	a. Switzerland	single center/Op	a: 330<br>c: 43.6	b: 83.6 (80.9-86.7)<br>d: Bern scale n.a.<br>MMSE<27 110 (33.3)<br>TUG≥20sec 113 (34.2)<br>MNA<12 154 (46.7)<br>BADL≥1 limitation 82 (24.9)<br>IADL≥1 limitation 218 (66.1)<br>Pre-clinical mob. disability 205 (62.1)	○ MMSE<br>○ TUG<br>○ MNA<br>○ BADL (Katz index)<br>○ IADL (Lawton index)<br>○ Pre-clinical mobility disability<br>○ Bern scale (from all above)	Long-	Low	6
Schoenenberger et al. 2013	a. Switzerland	single center/Op	a: 119<br>c: 44.5	b: 83.4 ± 4.6<br>d: Bern scale 59 (49.6)<br>MMSE<27points 39 (32.8)<br>TUG≥20sec 46 (38.7)<br>MNA<12point 53 (44.5)<br>BADL≥1 limitation 32 (26.9)<br>IADL≥1 limitation 72 (60.5)<br>Pre-clinical mob. disability 42 (35.3)	○ MMSE<br>○ TUG<br>○ MNA<br>○ BADL (Katz index)<br>○ IADL (Lawton index)<br>○ Pre-clinical mobility disability<br>○ Bern-scale (from all above)	Intermedate-	Moderate	7
Seiffert et al. 2014	a. Germany<br>c. Bonn TAVI registry	multicenter/Op	a: 845<br>c: 49	b: 80.9 ± 6.5<br>d: 16 (4.6)	○ CFS	Long-	High	5
Seiji et al. 2017	a. Japan<br>c. OCEAN-TAVI	multicenter/Or	a: 1.256<br>c: 28.7	a: < 80-years: 14.9%<br>80-84-y: 31.7%<br>85-89-y: 40.2%<br>≥ 90-y: 13.2%<br>d: Slow gait speed 429 (34)<br>Slowest gait speed 205 (16.2)<br>Unable to walk 59 (4.7)	○ Gait speed (5-mWT)<br>○ CFS<br>○ BMI<br>○ MMSE<br>○ Handgrip strength (dynamometer)<br>○ Serum albumin	Long-	High	4
Shi et al. 2019	a. USA, Canada, France<br>b. FRAILTY-AVR	multicenter/Or	a: 137<br>c: 48.2	b: 84.5 ± 5.8<br>d: overall prevalence of frailty phenotype 65	○ Composite index (according to Fried 5-domains)<br>○ CGA-FI (medical hystory, ADL, IADL, NAGI, Rosow-Breslau, MMSE, gait speed, handgrip strength, serum albumin, BMI, unintentional weight loss)	Intermediate-	Moderate	7
Shibata et al. 2018	a. Japan<br>c. OCEAN-TAVI	multicenter/Or	a: 1.613<br>c: 29.6	b: GNRI ≥92: 84 ± 5<br>GNRI 82-92: 85.1 ± 5.3<br>GNRI ≤82: 85.4 ± 4.9<br>d: GNRI ≤82: 132 (8.2)<br>GNRI 82-92: 396 (24.6)	○ GNRI	Long-	Moderate	7
Shimura et al. 2018	a. Japan	multicenter/Or	a: 1.542	b: Refusal: 87.3 ± 3.6	○ CFS	Short-	Moderate	7

	c. OCEAN-TAVI		c: 29.8	Non-refusal: 84.3 ± 5.1<br>d: Serum albumin 341 (22.1)<br>CFS n.a.<br>MMSE n.a.	○ MMSE<br>○ Serum albumin	Long-		
Shimura et al. 2017	a. Japan<br>c. OCEAN-TAVI	multicenter/Or	a: 1.215<br>c: 29.7	b: 84.4 ± 5.0<br>d: CFS score 4: 400 (32.9)<br>CFS score 5: 183 (15.1)<br>CFS score 6: 122 (10)<br>CFS score ≥7: 48 (4)	○ CFS<br>○ Gait speed (5-mWT)<br>○ Handgrip strength (dynamometer)	Short-	Moderate	7
Skaar et al. 2019	a. Norway	single center/Op	a: 142<br>c: 46	b: 83 ± 4<br>d: GA-Frailty score 34 (24%)<br>mEFT n.a.	○ GA-Fralty score (MMSE, NEDAL, BMI, SOF-index/energy/limb strength, modified SOF-index/weight loss, Chalson index, HADS)<br>○ mEFT (modified EFT)	Long-	Low	7
Steinvil et al. 2018	a. USA<br>c. MedStar Washington Hospital Center	single center/Or	a: 498<br>c: 49	b: 82 ± 8<br>d: Composite index 232 (46.6)	○ Gait speed (5-mWT)<br>○ Handgrip strength (dynamometer)<br>○ ADL (Katz index)<br>○ Serum albumin<br>○ BMI<br>○ Composite index (from all above)	Short-<br>Long-	High	5
Stortecky et al. 2012	a. Switzerland	single center/Op	a: 100<br>c: 40	b: 83.7 ± 4.6<br>d: Bern scale 49 (49)<br>MMSE<27 points 32 (32)<br>TUG<20sec 38 (38)<br>MNA<12 points 44 (44)<br>BADL>1 limitation 29 (29)<br>IADL>1 limitation 58 (58)<br>Pre-clinical mob. disability 60 (60)	○ MMSE<br>○ TUG<br>○ MNA<br>○ BADL (Katz index)<br>○ IADL (Lawton index)<br>○ Pre-clinical mobility disability<br>○ Bern-scale (from all above)	Short-<br>Long-	Low	9
Szekely et al. 2019	a. Israel	single center/Op	a: 1.029<br>c: RDW ≤15.5%: 41<br>RDW > 15.5%: 49	b: 83.1 ± 6.3<br>d: RDW ≤15.5%: 22<br>RDW > 15.5%: 27	○ Katz index	Long-	High	6
Tokuda et al. 2020	a. Japan<br>c. OCEAN-TAVI	multicenter/Or	a: 1.375<br>c: 29.2	b: Sarcopenia low CT: 84.9 ± 4.7<br>Sarcopenia high CT: 83.7 ± 5.7<br>Non Sarc. Low CT: 85.1 ± 4.5<br>Non Sarc.High CT: 84.5 ± 5.0	○ Skeletal muscle area<br>○ Skeletal muscle density	Short-<br>Long-	High	4

				d: Sarcopenia low CT 403 (29.3)<br>Sarcopenia high CT 399 (29)				
Ungar et al. 2018	a. Italy, Netherlands, Canada<br>c. CGA TAVI	multicenter/Or	a: 71<br>c: 38	b: 85.4 ± 2.9<br>d: n.a.	○ CGA-MPI (ADL, IADL, SPMSQ, CIRS, MNA, ESS, No. of drugs used, cohabitant status)<br>○ Silver code<br>○ SPPB	Intermediate-	High	6
van der Wulp et al. 2020	a. Netherlands	single center/Op	a: 511<br>c: 44.8	b: 80 (76-84)<br>d: MMSE <27: 139 (30.5)<br>BADL ≤19 point: 234 (47.2)<br>IADL <6 point: 172 (36.1)<br>Serum albumin/MNA 118 (26.01)<br>TUG/gait speed 180 (35.2)	○ Pat history<br>○ Hetero-anamnesis<br>○ Medication review<br>○ MMSE<br>○ BADL (Barthel index)<br>○ IADL (Lawton index)<br>○ TUG/gait speed<br>○ MNA/serum albumin	Long-	Moderate	7
van Mourik et al. 2019	a. Netherlands<br>c. AMC TAVI	single center/Op	a: 583<br>c: 45.3	b: 82.6 (78.1-85.8)<br>d: n.a	○ Psoas muscle area	Short-<br>Long-	High	5
van Mourik et al. 2019 (b)	a. Italy, Netherlands, Italy<br>c. CGA TAVI	multicenter/ Or	a: 71<br>c: 38	b: 85.4 ± 2.9<br>d: n.a.	○ MPI score<br>○ SPPB	Short-<br>Intermediate-<br>Long-	High	4
Yamamoto et al. 2015	a. France<br>c. FRANCE-2	multicenter/Op	a: 777<br>c: 48.5	b: BMI < 20: 85.0 ± 6.7<br>BMI 20-24.9: 84.0 ± 6.7<br>BMI ≥ 25: 82.7 ± 6.5<br>d: BMI < 20: 56 (7.2)	○ BMI	Short-<br>Long-	Low	7
Yokoyama et al. 2019	a. Japan	multicenter/Op	a: 767<br>c: 29	b: 84 ± 5<br>d: 554 (72)	○ CFS	Short-<br>Long-	High	5
